# Factors influencing home discharge after inpatient rehabilitation of older patients: a systematic review

**DOI:** 10.1186/s12877-016-0187-4

**Published:** 2016-01-12

**Authors:** Irma H. J. Everink, Jolanda C. M. van Haastregt, Sofie J. M. van Hoof, Jos M. G. A. Schols, Gertrudis I. J. M. Kempen

**Affiliations:** Department of Health Services Research, Faculty of Health, Medicine and Life Sciences and CAHPRI School for Public Health and Primary care, Maastricht University, Maastricht, The Netherlands; Department of Family Medicine, Faculty of Health, Medicine and Life Sciences and CAHPRI School for Public Health and Primary care, Maastricht University, Maastricht, The Netherlands

**Keywords:** Aged, Geriatrics, Rehabilitation, Patient discharge

## Abstract

**Background:**

Although rehabilitation for older patients has the potential to improve function and prevent admission to nursing homes, returning home after discharge is not possible for all patients. Better understanding of patient factors related to discharge home may lead to more realistic rehabilitation goals, more targeted rehabilitation interventions and better preparation of both patient and informal caregiver for discharge. Various studies provided insight into factors related to home discharge after stroke rehabilitation, but we still lack insight into factors related to home discharge in non-stroke patients. Therefore, the aim of this review is to provide an overview of factors influencing home discharge in older non-stroke patients admitted to an inpatient rehabilitation unit.

**Methods:**

A systematic literature search was executed in the databases PubMed, EMBASE, CINAHL and Web of Science to retrieve articles published between January 2000 and October 2015. The search focused on factors related to home discharge after rehabilitation for older patients. Studies were included if home discharge after rehabilitation was assessed as an outcome measure and if the non-stroke population was, on average, 65 years or older and admitted to an inpatient rehabilitation unit.

**Results:**

Eighteen studies were included. The methodological quality was moderate to good in 15 studies. The factors significantly associated with home discharge are younger age, non-white ethnicity, being married, better functional and cognitive status, and the absence of depression.

**Conclusions:**

Because various factors are significantly associated with home discharge of older non-stroke patients after rehabilitation, we recommend assessing these factors at admission to the rehabilitation unit. Further research into the factors that lack sufficient evidence concerning their association with home discharge is recommended.

**Electronic supplementary material:**

The online version of this article (doi:10.1186/s12877-016-0187-4) contains supplementary material, which is available to authorized users.

## Background

Hospitalization among older adults often results in functional decline and deterioration in self-care abilities [1]. Hospital stay is associated with inactivity and immobility, and prolonged hospital stay may have harmful effects such as muscle weakness, contractures and atrophy [2]. This impedes many community-dwelling older persons to return home directly after hospital discharge, especially frail patients with comorbidity and no family caregivers. In such cases, patients may be temporarily admitted to an inpatient rehabilitation unit. Such units use a multidisciplinary and comprehensive set of evaluative, diagnostic and therapeutic interventions focused on restoring functional capacity, activities of daily living and cognitive function [3, 4]. A study by Bachmann and colleagues revealed that rehabilitation among older patients has the potential to improve function, prevent permanent admission to nursing homes, and to decrease mortality [5].

Returning home is considered an indicator of successful rehabilitation and is frequently used as an indicator of quality of care [6, 7]. Yet, several studies have shown that a considerable number of older patients cannot return to their initial living arrangement after discharge from a rehabilitation unit, and have to be admitted to long-term care facilities [8–10].

Gaining more insight into the patient characteristics (measured at admission) related to returning to the initial living arrangement, may help care professionals to set more realistic rehabilitation goals and to prepare patients and informal caregivers for probable changes in their living arrangement after discharge [11]. Furthermore, increased insight into factors related to returning home may result in more accurate referrals to follow-up care after hospital discharge and therefore in a more efficient allocation of resources [5, 12, 13, 14].

In recent years, a substantial number of studies have been carried out to identify prognostic factors of home discharge after stroke rehabilitation [15–18]. Factors frequently found to be related to non-home discharge in stroke patients were older age, lower level of activities of daily living (ADL) functioning, the presence of cognitive disturbances and gender [15]. However, inpatient rehabilitation is also recommended for older patients with other medical conditions, such as those with Parkinson’s disease, amputation, arthritis, orthopedic disorders, chronic cardiac and pulmonary disease, and major multiple trauma. There is still a lack of insight into factors related to home discharge among this heterogeneous group of patients who often suffer from various comorbidities that influence the clinical course of their rehabilitation trajectory [19]. In contrast to stroke patients, non-stroke patients are more likely to be medically unstable: they are often admitted to the rehabilitation unit after trauma or an exacerbation of their illness and their rehabilitation trajectory is often complex. A better understanding in the factors related to home discharge might lead to establishing more realistic rehabilitation goals, tailored rehabilitation treatment, and a better preparation of patients and informal caregivers for the transition back home. Therefore, the purpose of the present study was to provide an overview of the factors influencing home discharge in older non-stroke patients admitted to an inpatient rehabilitation unit.

## Methods

### Search strategy

On the 15th of October 2015, a systematic search in four electronic databases (PubMed, EMBASE, CINAHL and Web of Science) was conducted. The search was focused on studies written in English published between 01-01-2000 and 15-10-2015. This timeframe was chosen to provide a realistic overview of the current situation in rehabilitation care for geriatric patients. Search terms used for the search strategy were the type of care, ‘rehabilitation’, combined with the Boolean operator ‘AND’ with search terms related to the rehabilitation setting (“rehabilitation unit” OR “rehabilitation center” OR “rehabilitation centre” OR “geriatric postacute rehabilitation” OR “geriatric post-acute rehabilitation” OR “intermediate care facilities” OR “skilled nursing facilities” OR “rehabilitation department” OR “inpatient rehabilitation” OR “department of rehabilitation” OR “rehabilitation ward”), the population (“aged”), the outcome measure (“discharge location” OR “living arrangements” OR “living setting” OR “independent living” OR “discharge destination” OR “home discharge” OR “community discharge”) and the focus of the research question (“determinant*” OR “prognos*” OR “indicator*” OR “influenc*” OR “predict*” OR “correlat*” OR “relat*” OR “prognosis” OR “associat*”). The full search strategy can be found in an additional file [see Additional file 1]. Additional studies were located based on the reference lists of the included studies.

### Study selection

Studies had to meet the following inclusion criteria:

- patients with a mean (or if not provided, a median) age of 65 years or older, who were admitted to an inpatient rehabilitation unit;
- factors potentially influencing discharge destination of these patients were measured within a week after admission to the rehabilitation unit;
- discharge location (home discharge versus non home discharge) was assessed as an outcome measure.

All studies that included patients who suffered from stroke were excluded from the review, also if the stroke patients only constituted a part of the study population. Furthermore, studies that only focused on a medical diagnosis as an influencing factor of home discharge were excluded from this review.

All literature results identified in the search were uploaded into EndNote. Two reviewers (authors IHJE and SJMvH) independently assessed abstracts to identify studies meeting the inclusion criteria for further review. In cases of disagreement, the study was included for full text review. All studies assessed as relevant were obtained in full text and reviewed independently by authors IHJE and SJMvH for definite inclusion according to the in- and exclusion criteria mentioned previously. In cases of disagreement, a third reviewer (author JCMvH) made the final decision on inclusion of studies based on the full text of the article.

### Data extraction and analysis

Using a structured data-extraction form, one author (IHJE) extracted data from the included studies. The primary outcome measure was home discharge. Furthermore, extracted data were study design, sample characteristics (i.e., sample size, age and gender), primary diagnosis, rehabilitation setting, discharge destination, effect size of influencing factor and interpretation. The effect sizes of the influencing factors were considered significant if they had a p-value ≤0.05. Data were categorized according to the factor that influenced home discharge.

In studies where multivariate statistical findings were presented, only these findings were extracted and incorporated into the data extraction table. In cases where only univariate statistical findings are included in the data extraction table this is an indication that the study did not display multivariate statistical findings.

### Methodological quality of identified studies

Quality appraisal of the included studies was independently done by authors IHJE and JCMvH using the checklist for quality assessment of prognostic studies developed by Hayden and colleagues [20]. In cases of disagreement, results were discussed until consensus was reached. This checklist comprised six domains (A-F; see Additional file 2) and each of the six domains was subdivided into three to seven items. The exact meaning of these items can be retrieved in an additional file [see Additional file 2]. The items were scored with *yes*, *partly*, *no*, *unsure* or *not applicable*. ‘Unsure’ was used when the item was relevant for the type of study design but not clearly described by the authors. ‘Not applicable’ was used when the item was irrelevant for the study design and was therefore not possible to be described by the authors.

A domain scored two points if all items in the domain scored ‘yes’, or if one item was scored with ‘partly’ and the other items within the domain were scored with ‘yes’. One point was allocated if the criteria necessary for receiving two points were not met but at least half of the items within the domain were scored with ‘yes’. If more than half of the items of the domain were scored with ‘partly’, ‘no’, or ‘unsure’, the domain was allocated zero points. If at least 90 % of the studies scored ‘not applicable’ on a specific item, that item was excluded from the domain.

Since there were six domains and a maximum of two points could be scored on each domain, the maximum possible score that could be gained was 12. The authors of the present review considered a score of 75 % (9 points) or higher to be a good methodological quality score. A score between 50–75 % (6–8 points) was considered a moderate methodological quality score whereas a score below 50 % (5 points or less) was considered a weak methodological quality score [21].

## Results

### Included studies

**Fig. 1:**
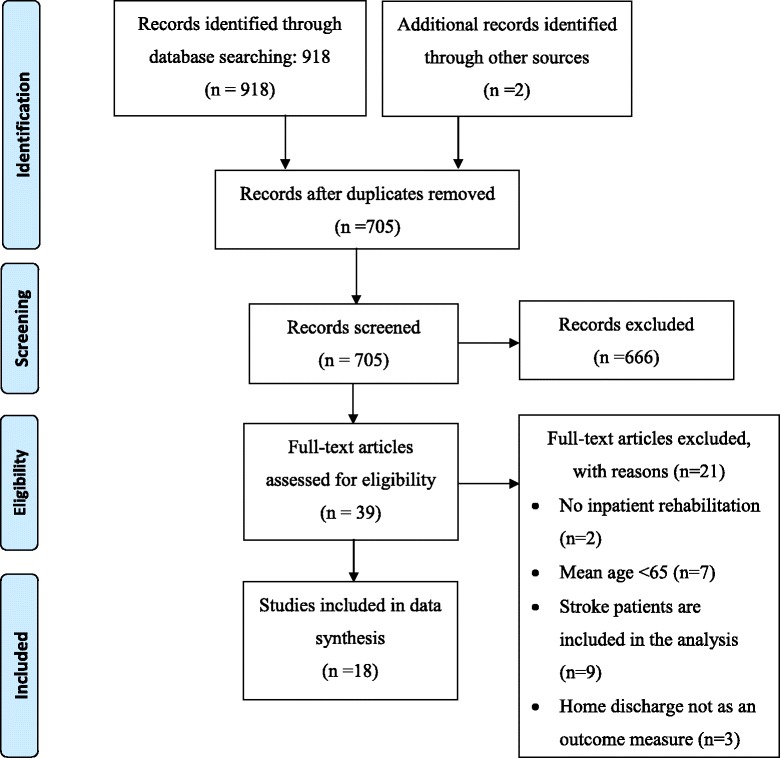
Flowchart of the record identification and selection process

Figure 1 shows the flowchart of the study identification and selection process. After removing duplicates, 705 potentially relevant articles were identified. Subsequently, after screening for title and abstract, 666 articles were excluded because they did not meet the inclusion criteria. The full texts of the remaining 39 articles were assessed, which led to the exclusion of another 21 studies. Thus, in total 18 articles were included in the review.

### Methodological quality

Table 1 shows the methodological quality of the 18 studies, based on the guidelines for assessing quality in prognostic studies by Hayden and colleagues [20]. The quality ranged from a score of 5 to 10 points (out of a theoretical range from 0 to 12), with a median of 7.5. After excluding the items that were not applicable in more than 90 % of the studies, domain A, ‘study participation’, consisted of five items. Domain B, ‘study attrition’, had one item, domain C, ‘prognostic factor measurement’, had five items, domain D, ‘outcome measurement’, had three items, domain E, ‘confounding measurement and account’, consisted of six items, and domain F, ‘analysis’, had three items.

**Table 1:** Methodological quality assessment

Author	Year	A^*^	B^**^	C	D	E	F	Total(12)
Berges [29]	2008	1	2	1	1	1	1	7
Chang [9]	2008	1	2	1	1	1	1	7
Chin [10]	2008	2	2	1	1	1	1	8
Graham [7]	2008	1	2	1	1	1	1	7
Hershkovitz [30]	2007	1	2	2	1	1	1	8
Kay [22]	2010	2	2	2	2	1	1	10
Kurichi [31]	2010	1	2	1	1	1	1	7
New [23]	2013	1	2	2	2	1	1	9
Sansone [27]	2007	1	2	2	2	1	1	9
Siebens [32]	2002	1	2	1	1	1	1	7
Vincent [33]	2006	1	2	2	1	0	1	7
Vincent [25]	2006	2	2	2	2	0	1	9
Vincent [26]	2006	2	2	1	2	1	1	9
Vincent [24]	2007	2	2	2	2	0	1	9
Vincent [34]	2008	0	2	1	0	0	2	5
Vincent [35]	2009	0	2	1	1	0	1	5
Vincent [14]	2010	0	2	1	1	0	1	5
Yan [28]	2013	1	2	2	2	1	1	9

*Domain A, ‘study participation’, consists of five items, domain B, ‘study attrition’, has one item, domain C, ‘prognostic factor measurement’, has five items, domain D, ‘outcome measurement’, has three items, domain E, ‘confounding measurement and account’, has six items and domain F, ‘analysis’, has three items

**In domain B, four items were not used in the calculation because they were not applicable in more than 90 % of the studies. In both domain C and in domain E, one item was not used in the calculation because it was not applicable in more than 90 % of the items

Seven studies [22–28] had a score of at least 75 % (9 points or more) of the total possible score of 12. Another eight studies [7, 9, 10, 29–33] scored 50–75 % (6 – 8 points) of the maximum score of 12, and three studies scored less than 6 points [14, 34, 35], which the authors of the present study considered of weak methodological quality.

The full quality appraisal on all 30 items can be found in an additional file [see Additional file 3].

### Data extraction

The characteristics of the studies are described in tables 2 and 3. Fifteen studies were conducted in the United States, one in Hong Kong, one in Israel and one in Australia. The sample sizes range from 119 to 63,793 participants. With one exception, all studies [31] included both male and female participants.

**Table 2:** Characteristics of identified studies

Author, year, Country [Reference Number]	Study design	Sample characteristics (n, age, gender)	Primary diagnosis	Rehabilitation setting	Discharge destination
Bergés et al., 2008, United States [29]	Retrospective cohort study	*n* = 63,793; mean age = 71.7; 66.5 % female	Hip replacement	Inpatient rehabilitation facilities	Home vs. not home
Chang et al., 2008, United States [9]	Retrospective cohort study	*N* = 9,240^a^; mean age = 78.8; 47 % female	Traumatic brain injury	Inpatient rehabilitation facilities	Home vs. not home
Chin et al., 2008; Hong Kong [10]	Prospective cohort study	*n* = 303, mean age = 82, 70 % female	Hip fracture	Inpatient rehabilitation hospital	Home vs. not home
Graham et al., 2008; United States [7]	Retrospective cohort study	*N* = 42,479; mean age = 82.2; 31.4 % female	Hip fracture	Inpatient rehabilitation facilities	Home vs. not home
Hershkovitz et al.,2007; Israel [30]	Prospective cohort study	*N* = 133; mean age = 80; 79.7 % female	Hip fracture	Rehabilitation unit of a geriatric hospital	Home vs. nursing home
Kay et al., 2010; United States [22]	Retrospective cohort study	*N* = 1,645, mean age = 70; 57.1 % female	Non-traumatic spinal cord injury	Inpatient rehabilitation facility	Residence vs. nursing home
Kurichi et al., 2013; United States [31]	Retrospective observational study	*N* = 1,480^b^, mean age = 66.8; 100 % male	Lower extremity amputation	Veterans Affairs Medical Centers	Home vs. not home
New, 2007; Australia [23]	Retrospective, 3-year case series	*N* = 70, mean age = 65; 54.3 % female	Non-traumatic spinal cord injury	Tertiary medical unit	Home vs. not home
Sansone et al., 2002; United States [27]	Retrospective study	*N* = 143, median age = 70; 39.9 % female	Cardiac patients	Public acute long-term care hospital	Home vs. not home
Siebens et al., 2012, United States [32]	Multi-site prospective observational cohort study	*N* = 224; mean age = 76.8; 78 % female	Hip fracture	9 skilled nursing facilities and 11 inpatient rehabilitation facilities	Home vs. not home
Vincent et al., 2006; United States [33]	Retrospective study	*N* = 332, mean age = 70.6; 63.6 % female	Total hip arthroplasty	Inpatient rehabilitation hospital	Home vs. not home
Vincent et al., 2006; United States [25]	Retrospective study	*N* = 424; mean age = 70.7; 70 % female	Total knee arthroplasty	Inpatient rehabilitation hospital	Home vs. not home
Vincent et al., 2006; United states [26]	Retrospective study	*N* = 402; mean age = 70.8; 62.9 % female	Total hip arthroplasty	Inpatient rehabilitation hospital	Home vs. not home
Vincent et al., 2007; United States [24]	Retrospective, comparative study	*N* = 146; mean age = 70.8; 70.1 % female	Total knee arthroplasty	Inpatient rehabilitation hospital	Home vs. not home
Vincent et al., 2008; United States [34]	Retrospective study	*N* = 23,649, mean age = 70.2; Female = 67 %	Total hip or knee arthroplasty	Inpatient rehabilitation facility	Home vs. not home
Vincent et al., 2009; United States [35]	Retrospective, comparative study	*N* = 1,947, mean age = 71; female = 70.5 %	Total hip arthroplasty	Inpatient rehabilitation facilities	Home vs. not home
Vincent et al., 2010; United States [14]	Multicenter, retrospective study	*N* = 5,421, mean age = 69.8; 68.6 % female	Total knee arthroplasty	15 Inpatient rehabilitation facilities	Home vs. not home
Yan et al., 2013; United States [28]	Retrospective study	*N* = 119; mean age = 67.4; 5.9 % female	Total knee arthroplasty/bilateral knee surgery/total hip arthroplasty	Inpatient rehabilitation in a Veterans Affairs Medical Center	Home vs. not home

vs. stands for ‘versus’. “Home” discharge means discharge to home, the community or an assisted living facility. Discharge to “not home” means discharge to a variety of inpatient care facilities, such as a skilled nursing facility, a nursing home, or acute care

^a^Before hospitalization, 8 % of the total population came from intermediate care or another hospital

^b^Before hospitalization, 1.5 % of the total population lived in an institution

**Table 3:** Data extraction of identified studies

Influencing factor	Study	Influencing factor specified	Discharge destination	Result	Interpretation
Age	Bergés et al., 2008 [29]	Higher age	Home vs. institution	OR = 0.97 (0.97–0.98)*	Higher age is related to fewer home discharges
	Chang et al., 2008 [9]	Each 1 year increase in age	Home vs. institution	OR = 0.99 (0.99–1.00)	Higher age is not related to discharge disposition
	Chin et al., 2008 [10]	Age ≥ 80 years	Institution vs. home	OR = 1.92 (1.04–3.57)*	Higher age is related to discharge to an institution
	New, 2007 [23]	Higher age	Home vs. institution	Wilkinson-rank sum test; *p* =0.01*	Higher age is related to fewer home discharges
	Sansone et al., 2002 [27]	Age	Home vs. institution	OR = 1.05 (0.99–1.09)	Age is not related to discharge disposition
	Siebens et al., 2012 [32]	Higher age	Home vs. institution	OR = 0.92 (0.87–0.96)*	Higher age is related to fewer home discharges
	Vincent et al., 2006 [33]	Age group <85 vs. ≥85	Home vs. institution	*χ* ^2^; *p* < 0.05*	Higher age is related to fewer home discharges
	Vincent et al., 2008 [34]	Age group <85 vs. ≥85	Non-home vs. home	OR = 3.63(3.37–3.89)‡	Higher age is related to fewer home discharges
	Yan et al., 2013 [28]	Younger age	Home vs. not home	MANOVA; *p* = 0.04*	Younger age is related to home discharge
Ethnicity	Bergés et al., 2008 [29]	Black vs. white	Home vs. institution	OR = 1.23 (1.07–1.41)*	Black race is related to home discharge
		Hispanic vs. white		OR = 1.51 (1.15–1.99)*	Hispanic race is related to home discharge
		Asian vs. white		OR = 1.67 (0.93–3.00)	Asian race is not related to discharge disposition
	Chang et al., 2008 [9]	Black vs. white	Home vs. institution	OR = 2.00 (1.55–2.59)*	Black race is related to home discharge
				OR = 2.24 (1.66–3.02)*	Hispanic race is related to home discharge
		Hispanic vs. white			
	Graham et al., 2008 [7]	Non-Hispanic black vs. white	Home vs. institution	OR = 2.02 (1.77–2.32)*	Non-Hispanic Black race is related to home discharge
		Hispanic vs. white		OR = 1.90 (1.64–2.19)*	Hispanic race is related to home discharge
		Asian vs. white		OR = 2.07 (1.55–2.78)*	Asian race is related to home discharge
	Siebens et al., 2012 [32]	Nonwhite vs. white	Home vs. institution	OR = 4.34 (0.86–21.79)	Nonwhite race is not related discharge disposition
Marital status	Bergés et al., 2008 [29]	Married vs. not-married	Home vs. institution	OR = 2.42 (2.24–2.61)*	Being married is related to home discharge
	Chang et al., 2008 [9]	Not-married vs. married	Home vs. institution	OR = 0.45 (0.40–0.51)*	Being not-married is related to fewer home discharges
	Kurichi et al., 2013 [30]	Married vs. not-married	Home vs. institution	OR = 1.51 (1.14–1.99)**	Being married is related to home discharge
Functional status	Chin et al., 2008 [10]	Admission FIM score <75	Institution vs. home	OR = 4.68 (2.23–9.82)*	Lower functional status at admission is related to discharge to an institution
	Sansone et al., 2002 [27]	Admission FIM score ≤71vs admission FIM score >72	Home vs. institution	OR = 0.91 (0.85–0.96)***	Lower functional status at admission is related to fewer home discharges
	Yan et al., 2013 [28]	Admission FIM score	Home vs. institution	MANOVA *p* = 0.00***	Higher functional status at admission is related to home discharge
Cognition	Chin et al., 2008 [10]	Admission AMT <6	Institution vs. home	OR = 1.60 (0.87–2.96)	Impaired cognitive function at admission is not related to discharge disposition
	Hershkovitz et al., 2007 [30]	MMSE score	Home vs. nursing home	OR = 1.11 (1.03–1.20)*	Higher cognitive functional level is related to home discharge
	Siebens et al., 2012 [32]	Cognitive FIM at admission	Home vs. institution	OR = 1.06 (1.01–1.11)*	Better cognitive function at admission is related to home discharge
Depression	Hershkovitz et al., 2007 [30]	Presence of depression	Home vs. nursing home	OR = 0.30 (0.11–0.84)*	The presence of depression is related to fewer home discharges
	Kurichi et al., 2013 [31]	Presence of depression	Home vs. institution	OR = 0.63 (0.40–0.98)*	The presence of depression is related to fewer home discharges
Clinical severity of illness	Siebens et al., 2012 [32]	Lower maximum severity	Home vs. institution	OR = 0.95 (0.93–0.97) ***	Lower severity of illness calculated by the CSI is related to home discharge
Treatment-level procedures	Kurichi et al., 2013 [31]	Ongoing active cardiac pathology (yes vs. no)	Home vs. institution	OR = 0.55 (0.37–0.81)**	Treatment level procedure is related to home discharge
Pre-hospital living situation	Yan et al., 2013 [28]	Lives alone vs. lives with someone	Home vs. institution	Fisher’s Exact Text: *p* = 0.35	Living alone is not related to discharge disposition
	Hershkovitz et al., 2007 [30]	Presence of a caregiver at home	Home vs. nursing home	OR = 8.88 (1.76–44.9)*	The presence of a caregiver at home is related to home discharge
Gender	Bergés et al., 2008 [29]	Male vs. female	Home vs. institution	OR = 1.08 (1.01–1.17)*	Male gender is related to home discharge
	Chang et al., 2008 [9]	Male vs. female	Home vs. institution	OR = 0.85 (0.75–0.96)*	Male gender is related to fewer home discharges
	Kay et al., 2010 [22]	Male vs. female; etiology degenerative spinal disorders	Community-based residence vs. nursing home	OR = 1.00 (0.50–1.99)	Gender is not related to discharge disposition
				OR = 0.98 (0.39–2.45)	Gender is not related to discharge disposition
		Male vs. female; etiology malignant tumor		OR = 0.73 (0.22–2.49)	Gender is not related to discharge disposition
		Male vs. female; etiology vascular ischemia			
	New, 2007 [23]	Male vs. female	Home vs. not home	*χ* ^2^; *p* = 0.00**	Female gender is related to fewer home discharges
	Sansone et al., 2002 [27]	Male vs. female	Home vs. institution	OR = 1.01 (0.35–2.95)	Gender is not related to discharge disposition
	Vincent et al., 2006 [33]	Female vs. male	Home vs. institution	*χ* ^2^; *p* < 0.05*	Female gender is related to fewer home discharges
	Yan et al., 2013 [28]	Male vs. female	Home vs. institution	Fisher’s Exact Test; *p* = 0.27	Male gender is not related to discharge disposition
Comorbidity	Berges et al., 2008 [29]	One or more	Home vs. institution	OR = 1.14 (0.83–1.57)	The presence of one or more comorbidities is not related to discharge disposition
	Chang et al., 2008 [9]	1–3 > 3	Home vs. institution	OR = 1.09 (0.73–1.63)	The presence of one or more comorbidities is not related to discharge disposition
				OR = 1.35 (0.95–1.93)	
	Chin et al., 2008 [10]	CVA or Parkinsonism	Institution vs. home	OR = 1.18 (0.56–2.51)	The presence of CVA or Parkinsonism as a comorbidity is not related to discharge disposition
	Kurichi et al., 2013 [31]	Congestive heart failure	Home vs. institution	OR = 0.62 (0.45–0.85)**	The presence of congestive heart failure as a comorbidity is associated with fewer home discharges
	Sansone et al., 2002 [27]	1 or more vs. 0	Home vs. institution	OR = 1.13 (0.37–3.38)	The presence of a comorbidity is not associated with discharge disposition
	Yan et al., 2013 [28]	Number of comorbidities	Home vs. institution	MANOVA *p* = 0.32	The number of comorbidities is not associated with discharge disposition
Type of surgery	Chin et al., 2008 [10]	Arthroplasty vs. Closed Reduction Internal Fixation (CRIF)	Institution vs. home	OR = 0.99 (0.56–1.73)	Fracture management is not related to discharge disposition
	Vincent et al., 2006 [26]	Home discharge in the primary total hip arthroplasty (THA) group vs. the revision THA group	Home vs. institution	Kruskal-Wallis; *p* < 0.00***	Type of surgery in hip arthroplasty patients is related to home discharge
	Vincent et al., 2006 [25]	Home discharge in the primary total knee arthroplasty (TKA) group vs. the revision TKA group	Home vs. institution	Kruskal-Wallis; *p* < 0.00***	Type of surgery in knee arthroplasty patients is related to home discharge
	Vincent et al., 2008 [34]	Bilateral joint procedures (THA + TKA) or unilateral joint procedures	Home vs. institution	OR = 0.76 (0.49–1.01)	The type of joint procedure is not related to discharge disposition
Postoperative complications	Chin et al., 2008 [10]	Chest infection or urinary tract infection	Institution vs. home	OR = 1.44 (0.56–3.69)	The postoperative complications chest infection or urinary tract infection are not related to discharge disposition
	Kurichi et al., 2013 [31]	Local significant infection at amputation	Home vs. institution	OR = 0.57 (0.39–0.83)**	Postoperative complications are related to fewer home discharges
Admission weight-bearing status	Siebens et al., 2012 [32]	Weight bearing as tolerated (WBAT) vs. restricted weight bearing (RWB) after hip fracture	Home vs. institution	OR = 2.58 (0.99–6.70)	Admission status “weight bearing as tolerated” is not related to discharge disposition
Hematocrit value	Vincent et al., 2010 [14]	Very low hematocrit (Hct <30 %) vs. low Hct (30–36 % women; 30–41 % men) vs. normal Hct (>36 % women; >41 % men)	Home vs. institution	*χ* ^2^; *p* > 0.05	Hematocrit value is not related to discharge disposition
Distance	Yan et al., 2013 [28]	Distance from inpatient rehabilitation facility in miles	Home vs. institution	MANOVA *p* = 0.09	The distance from the inpatient rehabilitation facility is not related to discharge disposition
Length of Stay in acute setting	Chin et al., 2008 [10]	>7 days	Institution vs. home	OR = 1.05 (0.59–1.87)	The length of stay in the acute setting is not related to discharge disposition
Obesity	Vincent et al., 2007 [24]	BMI <30 kg/m^2^ vs. BMI ≥30 kg/m^2^	Home vs. institution	*χ* ^2^; *p* >0.05	Obesity is not related to discharge disposition
	Vincent et al., 2008 [34]	BMI ≥ 50 kg/m^2^ vs. BMI <50 kg/m^2^	Home vs. institution	OR = 0.97 (0.71–1.23)	BMI is not related to discharge disposition
	Vincent et al., 2009 [35]	BMI <25 kg/m^2^ vs. BMI 25–29.9 kg/m^2^vs BMI 30–40 kg/m^2^ vs. BMI >40 kg/m^2^	Home vs. institution	*χ* ^2^; *p* >0.05	BMI is not related to discharge disposition
	Yan et al., 2013 [28]	Difference in BMI between home discharge and not home discharge	Home vs. institution	MANOVA *p* = 0.78	BMI is not related to discharge disposition
Pain	Chin et al., 2008 [10]	VAS pain scale at admission ≥4	Institution vs. home	OR = 0.61 (0.33–1.13)	Higher pain score at admission is not related to discharge disposition
Pre-fracture mobility status	Chin et al., 2008 [10]	Dependent or non-walker	Institution vs. home	OR = 1.84 (0.94–3.60)	Pre-fracture dependent mobility status is not related to discharge disposition
Pressure sore	Chin et al., 2008 [10]	Pressure sore at admission to rehabilitation	Institution vs. home	OR = 1.10 (0.44–2.73)	The presence of a pressure sore at admission is not related to discharge disposition
Primary insurance	Chang et al., 2008 [9]	Private vs. Medicare	Home vs. institution	OR = 1.01 (0.81–1.25)	The type of primary insurance is not related to discharge disposition
		Medicaid vs. Medicare		OR = 1.01 (0.45–2.28)	
		Other vs. Medicare		OR = 1.23 (0.70–2.17)	
Smoking history	Sansone et al., 2002 [27]	Smoker vs. non-smoker	Home vs. institution	OR = 3.17 (0.86–11.63)	Smoking history is not related to discharge disposition

vs. stands for versus; CVA denotes cerebrovascular accident; FIM Functional independence measure; AMT Abbreviated Mental Test; MMSE Mini Mental State Examination; CSI Comprehensive Severity Index and VAS Visual Analogue Scale

*P < 0.05

**p < 0.01

***p < 0.001

### Factors influencing home discharge after inpatient rehabilitation

Twenty-four factors that potentially influenced discharge destination were identified (Table 3). Seven out of nine studies found a significant relationship between higher age and non-home discharge after inpatient rehabilitation [10, 23, 28, 29, 32–34]. The influence of ethnicity on home discharge was assessed in four studies. Three studies demonstrated that black and Hispanic ethnicity were significantly related to higher percentages of home discharge, compared to their white counterparts [7, 9, 29] and one study did not report a significant relationship between ethnicity and home discharge [32]. Three studies investigated the association between marital status and discharge disposition. All of these studies revealed that being married is significantly related to home discharge [9, 29, 31]. Three studies indicated a positive association between higher functional status at admission and home discharge [10, 27, 28]. Furthermore, better cognitive function at admission was significantly related to home discharge in two out of three studies [30, 32] and the presence of depression at admission was significantly related to discharge to a facility rather than home, which was shown by two studies [30, 31].

The relationship between living situation (alone or with someone else) and home discharge was assessed in two studies. One study [30] found a significant relationship between having a caregiver at home and home discharge, whereas the other study did not find such an association between living alone and home discharge compared with living with someone else [28] Four out of seven studies found a significant relationship between gender and home discharge after inpatient rehabilitation. Three studies reported a significant relationship between male gender and home discharge [23, 29, 33], while one study revealed that being male is significantly related to non-home discharge [9].

Five out of six studies demonstrated the absence of a significant relationship between comorbidity and discharge destination [9, 10, 27–29] while one study claimed a negative significant relationship between congestive heart failure as a comorbid disease and home discharge [31]. An exception with respect to comorbidity is the influence of obesity on home discharge, which was examined in four studies. None of the four studies demonstrated a significant relationship between obesity and discharge destination [24, 28, 34, 35].

## Discussion

The findings from this systematic review show that home discharge after inpatient rehabilitation for geriatric patients is significantly related to younger age [10, 23, 28, 29, 32–34], non-white ethnicity [7, 9, 29], being married [9, 29, 31], higher functional [10, 27, 28] and cognitive [30, 32] status and the absence of depression [30, 31]. All predicting factors were measured at admission to the rehabilitation unit. Less clinical severity of the illness [32] and no active cardiac pathology [31] appeared to be significantly related to home discharge, however, these associations all come from only one study, therefore these results have to be treated with caution.

Due to inconsistent results, the association between home discharge and gender [9, 22, 23, 27–29, 33], comorbidity [9, 10, 29, 31], type of surgery [10, 25, 26, 34], living alone [28, 30] and postoperative complications [10, 31] was less obvious. These opposing outcomes might have been caused by differences in study populations (traumatic brain injury [9], hip replacement [10, 29, 30, 33], knee replacement [25, 28], spinal cord injury [23] and lower extremity amputation [31]) or a difference in the size of the study population [36]. Further research is required to explore the impact of these factors on home discharge after inpatient rehabilitation. In addition, no significant association was found between obesity and discharge disposition [24, 28, 34, 35]. The association between home discharge and the factors weight-bearing status at admission (restricted or not) [32], hematocrit value [14], travel distance from the inpatient rehabilitation facility [28], length of stay in the acute setting [10], pain [10], pre-fracture mobility status [10], the presence of a pressure sore [10], primary insurance [9], and smoking history [27] were also not significant. Because the evidence of these non-significant associations was based on single studies, further research into the impact of these factors is required. The three studies with weak methodological quality examined the association of higher age [34], type of surgery [34], Body Mass Index [34, 35] and hematocrit value [14] with home discharge. These effects might therefore also be treated with caution.

### Discriminative ability of methodological quality assessment domains

The methodological quality of 15 out of 18 studies could be defined as moderate to good. However, the discriminative ability of four domains with respect to methodological quality is questionable. After excluding items that were ‘not applicable’ in at least 90 % of the studies, domain B, ‘study attrition’, had only one item remaining. As a consequence, the score gained on that domain only ranged from 0 to 2. Since all included studies scored 2 points, this domain had no discriminative ability. The same holds for domain F focused on ‘analysis’. Although this domain consisted of three items, all studies had a score of 1, which again indicates a lack of discriminative ability. Furthermore, the scores on domain C, ‘prognostic factor measurement’, and domain E, ‘confounding measurement and account’, did not vary more than one point. It seems that, although assessing the methodological quality of the studies is done to differentiate between the quality of the included studies, some domains add very little to quality differences.

### Comparison with other research

The findings from the present systematic review are in line with several prognostic factors for non-home discharge in stroke patients, as the review of Meijer and colleagues showed [15]. This latter review found that low initial activities of daily living (ADL) functioning, high age, cognitive disturbance, and being female predicted less home discharge in the sub-acute phase after stroke [15]. Other factors associated with home discharge were stroke-related factors such as paresis of arm and leg, initial level of consciousness being ‘not alert’ and constructional apraxia; therefore, these results cannot be compared with the results of the present review.

Factors affecting discharge destination in older medical patients who return home after hospital admission without inpatient rehabilitation are also comparable as presented in a systematic literature review by Campbell and colleagues [37]. Their review showed significant findings for functional status, cognitive functioning and age in relation to discharge destination. Gender and comorbidity appeared to have no significant relationship with discharge destination [37].

Although this review revealed that ethnicity seems to have a significant influence on home discharge, ethnicity is not addressed in the reviews from Meijer and colleagues [15] and Campbell and colleagues [37].

### Issues to be considered

Some issues in this study need to be considered. First, we included studies with various patient populations. Although this is a good reflection of the heterogeneous population in rehabilitation, it is a methodological challenge because this hampers the comparability of the studies, and it is not clear whether a relationship observed in a specific diagnosis group will also be present in another diagnosis group. For this reason, we performed a subgroup analysis among the 13 studies that included only patients with orthopedic disorders. When analyzing the factors influencing home discharge among this subgroup, younger age, non-white ethnicity, higher functional and cognitive status still appear to be of significant value (the results are supported by at least two studies). The statistical significant effects of marital status and the absence of depression on home discharge are both supported by only one study in this subgroup analysis, and should therefore be treated with caution. This implies that, although minor differences exist, the factors influencing home discharge among the different diagnosis groups seem to be fairly comparable and may therefore be interpreted as rather robust. Apart from ethnicity, these results are also in line with influencing factors of home discharge among the stroke population [15].

Overall, our review found 23 possible influencing factors of home discharge after inpatient rehabilitation for geriatric patients but only six factors demonstrated a clear significant and rather consistent association. Therefore, future research into the inconsistent factors and into the factors that were only examined by one study is warranted.

### Study limitations

First, the quantity, intensity and quality of therapies offered within inpatient rehabilitation for older patients might differ between countries and between rehabilitation units, the received therapy was not described in the included studies and could therefore not be taken into account in this review. Despite the differences in the included studies in diagnosis, received therapy and admission rules, several predicting factors were rather similar across patients and settings thus showing their robustness as well.

Second, the validity of systematic reviews is dependent on the absence of publication bias [38]. The presentation of only those results that are significant with non-significant results being excluded from publication, could lead to misleading conclusions. Therefore, the risk of publication bias should always be taken into account when results are interpreted. Third, there is always a risk of missing studies because they were not identified by the search strategy. We tried to minimize this potential bias by not only screening articles identified by the databases, but by analyzing reference lists of included articles as well.

Another limitation of our study is that the data extraction has been conducted by one researcher instead of two researchers independently, which could affect rigor. Furthermore, analytic strategies in the included studies varied; both multivariate and univariate outcomes are presented. Although this is accounted for in the methodological quality assessment, it means that some studies adjusted for confounders while others did not.

Finally, the protocol of our study has not been registered or published. Because the methods used did not change during the course of the study, we believe that this did not affect our results.

## Conclusions

To help care professionals in setting more realistic rehabilitation goals and in preparing patients and informal caregivers for probable changes in living arrangement after discharge, we recommend assessing at least the following factors during admission of older patients to a rehabilitation unit: age, marital status, presence of depression, level of cognitive functioning and functional status. This assessment will help care professionals to make a more reliable prediction of discharge destination and to optimally tailor the rehabilitation treatment to the needs of the patient and their family. Because the prognostic factors of home discharge among stroke patients appear to be comparable to those of non-stroke patients, this assessment can be applied to all older patients admitted to an inpatient rehabilitation unit.

## Additional files

Additional file 1:
**Search strategy.** (DOCX 15 kb) Additional file 2:
**Methodological quality assessment items.** (DOCX 18 kb) Additional file 3:
**Methodological quality assessment of included studies.** (DOCX 28 kb)
