# Supplementary material for: Factors influencing home discharge after inpatient rehabilitation of older patients: a systematic review
Source: BMC Geriatr. 2016 Jan 12;16:5. doi: 10.1186/s12877-016-0187-4 (PMC4709872; doi:10.1186/s12877-016-0187-4)
Supplement: Additional file 1: — Search strategy. (DOCX 15 kb) [file 12877_2016_187_MOESM1_ESM.docx]

**Additional file 1: Search strategy**

PubMed:

((((determinant* OR Prognos* OR indicator* OR influenc* OR predict* OR correlat* OR Relat* OR “prognosis”[Mesh] OR associat*) AND (“Aged”[Mesh] OR “Aged, 80 and over”[Mesh]) AND (“rehabilitation”[Mesh]) AND (“rehabilitation unit” OR “rehabilitation centre” OR geriatric postacute rehabilitation OR geriatric post-acute rehabilitation OR “Intermediate care facilities”[Mesh] OR “skilled nursing facilities”[Mesh] OR “rehabilitation department” OR inpatient rehabilitation OR “department of rehabilitation” OR “rehabilitation centers”[Mesh] OR “rehabilitation ward”))) AND (“discharge location” OR “living arrangements” OR “living setting” OR “independent living” OR “discharge destination” OR “home discharge” OR “community discharge” OR “discharge disposition”)

EMBASE:

(rehabilitation.mp) AND (rehabilitation center\ OR inpatient rehabilitation.mp OR skilled nursing facilities.mp OR rehabilitation department.mp OR rehabilitation ward.mp OR department of rehabilitation.mp OR intermediate care facilities.mp OR geriatric postacute rehabilitation.mp OR geriatric post-acute rehabilitation.mp OR rehabilitation centre.mp) AND (aged\) AND (relat*.mp OR associat*.mp OR prognosis\ OR predict*.mp OR correlat*.mp OR influenc*.mp OR prognos*.mp OR indicator*.mp OR determinant*.mp) AND (discharge location.mp OR living arrangements.mp OR living setting.mp OR independent living.mp OR discharge destination.mp OR home discharge.mp OR community discharge.mp OR discharge disposition.mp)

CINAHL:

(Rehabilitation) AND (“rehabilitation center[MH]” OR “inpatient rehabilitation” OR “rehabilitation centre” OR “skilled nursing facilities[MH]” OR “rehabilitation department” OR “rehabilitation ward” OR “department of rehabilitation” OR intermediate care facilities OR geriatric postacute rehabilitation OR geriatric post-acute rehabilitation) AND (“Aged[MH]”) AND (relat* OR associat* OR “prognosis[MH]” OR predict* OR correlat* OR influenc* OR prognos* OR indicator* OR determinant*) AND (“discharge location” OR “living arrangements” OR “living setting” OR “independent living” OR “discharge destination” OR “home discharge” OR “community discharge” OR “discharge disposition”)

Web of Science:

(TS=Rehabilitation) AND (TS=“rehabilitation center” OR TS=“inpatient rehabilitation” OR TS=“skilled nursing facilities” OR TS=“rehabilitation department” OR TS=“rehabilitation ward” OR TS=“department of rehabilitation” OR TS=intermediate care facilit* OR TS=geriatric postacute rehabilitation OR TS=geriatric post-acute rehabilitation OR TS=“rehabilitation centre”) AND (TS=Aged) AND (TS=relat* OR TS=associat* OR TS=prognosis OR TS=predict* OR TS=correlat* OR TS=influenc* OR TS=prognos* OR TS=indicator* OR TS=determinant*) AND ((TS=“discharge location” OR TS=“living arrangements” OR TS=“living setting” OR TS=“independent living” OR TS=“discharge destination” OR TS=“home discharge” OR TS=“community discharge” OR TS=“discharge disposition”)
