# Supplementary material for: Factors influencing home discharge after inpatient rehabilitation of older patients: a systematic review
Source: BMC Geriatr. 2016 Jan 12;16:5. doi: 10.1186/s12877-016-0187-4 (PMC4709872; doi:10.1186/s12877-016-0187-4)
Supplement: Additional file 2: — Methodological quality assessment items. (DOCX 18 kb) [file 12877_2016_187_MOESM2_ESM.docx]

**Appendix 2 Methodological quality assessment items**

| Study participation (A) | 1 | The source population or population under interest is adequately described for key characteristics. |
| --- | --- | --- |
|  | 2 | The sampling frame and recruitment are adequately described. |
|  | 3 | Inclusion and exclusion criteria are adequately described. |
|  | 4 | There is adequate participation in the study by eligible individuals. |
|  | 5 | The baseline study sample is adequately described for key characteristics. |
| Study attrition (B) | 6 | Response rate is adequate. |
|  | 7 | Attempts to collect information on participants who dropped out of the study are described. |
|  | 8 | Reasons for loss to follow-up are provided. |
|  | 9 | Participants lost to follow-up are adequately described for key characteristics. |
|  | 10 | There are no important differences between key characteristics and outcomes in participants who completed the study and who did not. |
| Prognostic factor measurement (C) | 11 | A clear definition or description of the prognostic factor measured is provided. |
|  | 12 | Continuous variables are reported or appropriate cut-points are used. |
|  | 13 | The prognostic factor measure and method are adequately valid and reliable to limit misclassification bias. |
|  | 14 | Adequate proportion of the study sample has complete data for prognostic factors. |
|  | 15 | The method and setting of measurement are the same for all study participants. |
|  | 16 | Appropriate methods are used if imputation is used for missing prognostic factor data. |
| Outcome measurement (D) | 17 | A clear definition of the outcome of interest is provided, including duration of follow-up and level and extent of the outcome construct. |
|  | 18 | The outcome measure and method used are adequately valid and reliable to limit misclassification bias. |
|  | 19 | The method and setting of measurement are the same for all study participants. |
| Confounding measurement and account (E) | 20 | All important confounders, including treatments, are measured. |
|  | 21 | Clear definitions of the important confounders measured are provided. |
|  | 22 | Measurement of all important confounders is adequately valid and reliable. |
|  | 23 | The method and setting of confounding measurement are the same for all study participants. |
|  | 24 | Appropriate methods are used if imputation is used for missing confounder data. |
|  | 25 | Important potential confounders are accounted for in the study design. |
|  | 26 | Important potential confounders are accounted for in the analysis. |
| Analysis (F) | 27 | There is sufficient presentation of data to assess the adequacy of the analysis. |
|  | 28 | The strategy for model building is appropriate and is based on a conceptual framework or model. |
|  | 29 | The selected model is adequate for the design of the study. |
|  | 30 | There is no selective reporting of results. |
