# Supplementary material for: Factors influencing home discharge after inpatient rehabilitation of older patients: a systematic review
Source: BMC Geriatr. 2016 Jan 12;16:5. doi: 10.1186/s12877-016-0187-4 (PMC4709872; doi:10.1186/s12877-016-0187-4)
Supplement: Additional file 3: — Methodological quality assessment of included studies. (DOCX 28 kb) [file 12877_2016_187_MOESM3_ESM.docx]

**Appendix 3: Methodological quality assessment of included studies**

| Domain | Study participation | | | | | Study attrition | | | | | Prognostic factor measurement | | | | | | Outcome measurement | | | Confounding measurement and account | | | | | | | Analysis | | | |
| --- | --- | --- | --- | --- | --- | --- | --- | --- | --- | --- | --- | --- | --- | --- | --- | --- | --- | --- | --- | --- | --- | --- | --- | --- | --- | --- | --- | --- | --- | --- |
| Item | **1** | **2** | **3** | **4** | **5** | **6** | **7** | **8** | **9** | **10** | **11** | **12** | **13** | **14** | **15** | **16** | **17** | **18** | **19** | **20** | **21** | **22** | **23** | **24** | **25** | **26** | **27** | **28** | **29** | **30** |
| Berges et al., 2008[29] | **±** | **±** | **+** | **+** | **+** | **+** | **na** | **na** | **na** | **na** | **+** | **+** | **+** | **+** | **?** | **na** | **+** | **+** | **?** | **±** | **+** | **+** | **?** | **na** | **+** | **+** | **+** | **?** | **+** | **+** |
| Chang et al., 2008[9] | **±** | **±** | **+** | **+** | **+** | **+** | **na** | **na** | **na** | **na** | **+** | **+** | **+** | **+** | **?** | **na** | **+** | **+** | **?** | **±** | **+** | **+** | **?** | **na** | **-** | **+** | **+** | **?** | **+** | **+** |
| Chin et al., 2008[10] | **+** | **+** | **+** | **+** | **±** | **+** | **na** | **na** | **na** | **na** | **+** | **+** | **+** | **+** | **?** | **na** | **+** | **+** | **?** | **±** | **+** | **+** | **?** | **na** | **-** | **+** | **+** | **?** | **+** | **+** |
| Graham et al., 2008[7] | **-** | **+** | **+** | **+** | **+** | **+** | **na** | **na** | **na** | **na** | **+** | **+** | **+** | **+** | **?** | **na** | **+** | **+** | **?** | **±** | **+** | **+** | **?** | **na** | **-** | **+** | **+** | **?** | **+** | **+** |
| Hershkovitz et al., 2007[30] | **-** | **+** | **±** | **+** | **+** | **+** | **na** | **na** | **na** | **na** | **+** | **+** | **+** | **+** | **+** | **na** | **±** | **+** | **+** | **+** | **+** | **+** | **+** | **na** | **-** | **+** | **+** | **?** | **+** | **?** |
| Kay et al., 2010[22] | **+** | **+** | **+** | **+** | **+** | **+** | **na** | **na** | **na** | **na** | **+** | **+** | **+** | **+** | **+** | **na** | **+** | **+** | **?** | **±** | **+** | **+** | **?** | **+** | **-** | **+** | **+** | **?** | **+** | **+** |
| Kurichi et al., 2013[31] | **-** | **+** | **+** | **+** | **+** | **+** | **na** | **na** | **na** | **na** | **+** | **?** | **+** | **+** | **?** | **na** | **+** | **+** | **?** | **+** | **+** | **+** | **?** | **na** | **-** | **+** | **+** | **?** | **+** | **+** |
| New, 2007[23] | **-** | **+** | **+** | **+** | **+** | **+** | **-** | **+** | **-** | **?** | **+** | **+** | **+** | **+** | **+** | **na** | **±** | **+** | **+** | **+** | **+** | **+** | **+** | **na** | **-** | **-** | **+** | **?** | **+** | **+** |
| Sansone et al., 2002[27] | **+** | **+** | **±** | **?** | **+** | **+** | **na** | **na** | **na** | **na** | **+** | **+** | **+** | **+** | **+** | **na** | **+** | **+** | **+** | **±** | **+** | **+** | **+** | **na** | **-** | **+** | **+** | **?** | **+** | **+** |
| Siebens et a l., 2012[32] | **±** | **+** | **±** | **+** | **+** | **+** | **na** | **na** | **na** | **na** | **±** | **+** | **+** | **+** | **?** | **na** | **+** | **+** | **?** | **±** | **+** | **+** | **?** | **na** | **-** | **+** | **+** | **?** | **+** | **+** |
| Vincent et al., 2006[33] | + | **±** | **±** | **+** | **+** | **+** | **na** | **na** | **na** | **na** | **+** | **+** | **+** | **+** | **+** | **na** | **+** | **+** | **?** | **±** | **±** | **+** | **+** | **na** | **-** | **-** | **+** | **?** | **±** | **+** |
| Vincent et al., 2006[25] | + | **+** | **±** | **+** | **+** | **+** | **na** | **na** | **na** | **na** | **+** | **+** | **+** | **+** | **+** | **na** | **+** | **+** | **+** | **±** | **±** | **?** | **+** | **na** | **-** | **-** | **+** | **?** | **±** | **+** |
| Vincent et al., 2006[26] | **+** | **+** | **±** | **+** | **+** | **+** | **na** | **na** | **na** | **na** | **+** | **-** | **+** | **+** | **+** | **na** | **+** | **+** | **+** | **±** | **+** | **+** | **+** | **na** | **-** | **-** | **+** | **?** | **±** | **+** |
| Vincent et al., 2007[24] | **+** | **+** | **±** | **+** | **+** | **+** | **na** | **na** | **na** | **na** | **+** | **+** | **+** | **+** | **+** | **na** | **+** | **+** | **+** | **±** | **±** | **+** | **+** | **na** | **-** | **-** | **+** | **?** | **±** | **+** |
| Vincent et al, 2008[34] | **±** | **±** | **±** | **+** | **+** | **+** | **na** | **na** | **na** | **na** | **+** | **+** | **+** | **+** | **?** | **na** | **±** | **?** | **?** | **±** | **±** | **±** | **?** | **na** | **-** | **+** | **+** | **?** | **+** | **+** |
| Vincent et al., 2009[35] | **±** | **±** | **±** | **+** | **+** | **+** | **na** | **na** | **na** | **na** | **+** | **+** | **+** | **+** | **?** | **na** | **+** | **+** | **?** | **-** | **na** | **na** | **na** | **na** | **-** | **-** | **+** | **?** | **±** | **+** |
| Vincent et al., 2010[14] | **±** | **±** | **±** | **+** | **+** | **+** | **na** | **na** | **na** | **na** | **+** | **+** | **+** | **+** | **?** | **na** | **+** | **+** | **?** | **±** | **na** | **na** | **na** | **na** | **-** | **-** | **+** | **?** | **±** | **+** |
| Yan et al., 2013[28] | **+** | **±** | **±** | **+** | **+** | **+** | **na** | **na** | **na** | **na** | **+** | **+** | **+** | **±** | **+** | **na** | **+** | **+** | **+** | **±** | **+** | **+** | **+** | **na** | **-** | **+** | **+** | **?** | **+** | **+** |

**‘+’ = item is described; ‘±’ = item is partly described; ‘-‘ = item is not described; ‘?’ = unknown; ‘na’ = item is irrelevant for the study design and therefore not described.**
